# Knowledge and Attitudes of Parents about Oral Health in the Primary Dentition Stage in a Peruvian High Andean City

**DOI:** 10.3390/ijerph21020154

**Published:** 2024-01-30

**Authors:** Nilton B. Rojas-Briceño, Oscar J. Oc Carrasco, Yshoner A. Silva Díaz, Carla M. Ordinola Ramírez, Oscar Pizarro Salazar, Shírley J. Tuesta-Mendoza, Jhonsy O. Silva-López

**Affiliations:** 1 Facultad de Ciencias de la Salud, Universidad Nacional Toribio Rodríguez de Mendoza de Amazonas, Chachapoyas 01001, Peru; oscar.oc@untrm.edu.pe (O.J.O.C.); carla.ordinola@untrm.edu.pe (C.M.O.R.);; 2 Escuela Profesional de Ingeniería Ambiental, Facultad de Ingeniería y Arquitectura, Universidad Nacional de Moquegua, Moquegua 18610, Peru

**Keywords:** dental caries, dental health, oral habits, preschool, primary teeth, thumb sucking

## Abstract

Children’s oral health depends on parents’ knowledge and attitudes. The primary dentition stage, in particular, plays a crucial role in the comprehensive development of children. Therefore, the objective was to evaluate parents’ knowledge and attitudes about oral health in the primary dentition stage in Chachapoyas (Amazonas, NW Peru). A questionnaire was administered to 409 parents from 15 educational institutions, and the data were analyzed using multinomial logistic regression. Among the surveyed parents, 75.3% could identify at least one habit harmful to their children’s teeth (such as excessive sugar consumption) and one of its effects (such as dental caries). Additionally, 77.5% reported that their children presented some dental problem, more frequent in the peripheral areas than in the city’s center. Gender (odd ratio, OR = 0.484; *p* = 0.037), educational level (OR = 2.144; *p* = 0.043), and type of health insurance (OR = 2.627; *p* = 0.044) of the parents influenced awareness of taking care of their children’s primary dental health. The geographic location of the school (1.729 < OR < 2.079; *p* ≤ 0.011) and family income (OR = 3.504; *p* = 0.028) influenced parents’ identification of the different harmful effects of children’s habits. Factors such as low socioeconomic status and never taking the child to the dentist increased the risk of dental problems (*p* < 0.05). It is suggested that peripheral areas, like rural areas, lack the same oral health programs and access to treatment as central urban areas, leading to disparities in parental knowledge and attitudes.

## 1. Introduction

Primary dentition (baby teeth or milk teeth) is a fundamental stage for the integral development of children, impacting their physical growth, oral function, and psychosocial aspects [1]. However, previous research has determined that some parents believe that treating these teeth is unnecessary, as they consider them a natural loss as the child grows [2,3]. One of the most frequent causes of premature loss of primary teeth is dental caries [4], an oral disease considered an essential public health problem [5], with about 514 million cases and a global average prevalence of 43% [6]. Severe caries of primary teeth harms the development of permanent teeth and children’s oral and general health [7].

Several factors are related to the occurrence and development of childhood caries, such as some dietary patterns [8], frequency of sugary food intake [9,10], brushing habits [11], parents’ educational level [12], and family economic status [12,13,14]. Parents’ academic level and family financial status are factors that are difficult to change, but behaviors and lifestyles related to children’s oral health can be controlled by behavioral interventions [15]. Children’s oral health depends on parental knowledge and attitudes [2]. Parents are critical to their children’s oral health as primary caregivers and decision-makers [3].

Previous studies on parents’ knowledge, attitudes, and practices regarding their children’s primary dentition have identified that, in general, parents have a superficial or partial knowledge of primary teeth [3]. This is reflected in poor care practices for their children’s primary teeth [16]. On the other hand, mothers have a higher level of knowledge and a more positive attitude towards their children’s primary dentition health than fathers [17]. In addition, parents of high socioeconomic status are comparatively more knowledgeable about children’s primary teeth than parents of middle socioeconomic status [18]. Also, the urban population of parents of child patients seeking primary teeth treatment is larger than the rural population [19].

In Peru, the prevalence of untreated caries in primary teeth of children aged 1–9 years was estimated at 41.4–45.8%, whereas, in permanent teeth of persons ≥5 years, it was 35.6–40.6% in 2019 [6,20]. Previous studies found that parents’ knowledge, attitudes, and practices regarding their children’s oral health are fair, based on three categories (poor, fair, and good knowledge) [21]. In addition, a moderate but significant relationship has been established between inadequate parental attitudes and children’s oral health status [22]. A relationship has also been observed between oral health literacy and knowledge about harmful oral habits [23]. These studies do not focus on primary dentition; they have been conducted in large cities on the Peruvian coast [21,22,23,24,25,26] and some southern Andean cities [27,28,29], specific educational institutions, or specific health centers. However, documented research on parental knowledge, attitudes, and practices about primary dentition is limited, even more so in high Andean cities in the Amazonian regions of northern Peru.

Therefore, the aim of this study was to evaluate the knowledge and attitudes of parents about oral health in the primary dentition stage in the high Andean city of Chachapoyas, department of Amazonas, northern Peru. To achieve this aim, the objectives were to design, validate, and apply a questionnaire to parents, comparing the central area and the periphery (where the rural migrant population of the city is mainly concentrated). The purpose of this research is to fill a gap in the scientific literature and provide relevant information for the design of effective interventions to promote better oral health in the children of this city.

## 2. Materials and Methods

### 2.1. Study Area

**Figure 1:**
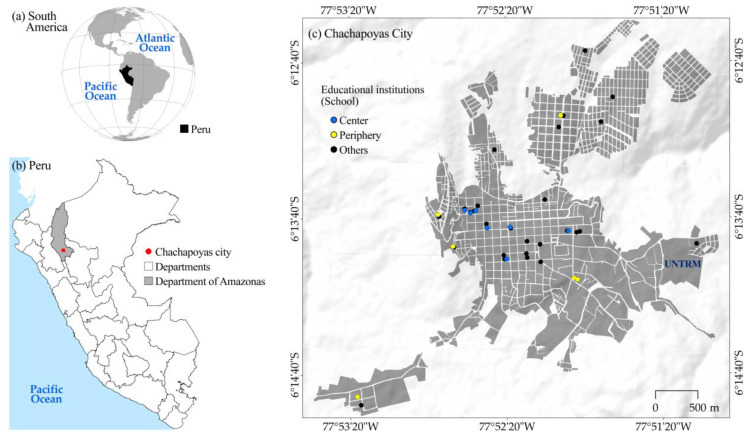
Study areas in Amazonas, northern Peru.

The city of Chachapoyas, administrative center (capital) of the Department of Amazonas, is located in the northeastern Andes of Peru, at 2483 m above sea level (Figure 1). Chachapoyas is the second most populated city in the department, with high economic, commercial, and tourist activity, and is expanding at an accelerated rate into new peripheral areas [30]. This is influenced by the migration of the rural population from the same department and other adjacent departments in search of higher education since the creation of the Universidad Nacional Toribio Rodríguez de Mendoza de Amazonas (UNTRM) in 2000. As a result, the population of Chachapoyas increased from 23,202 to 32,026 inhabitants between the 2007 and 2017 censuses [31], and currently, the population is estimated to be close to 40,000 [32].

### 2.2. Design, Population, Sample, and Sampling

A descriptive cross-sectional study design with a quantitative approach was used [33]. This study included parents from educational institutions at the primary level (6–11 years) and kindergarten (3–5 years). It was conducted at the level of educational institutions because they concentrate a large number of children in the primary dentition stage, providing a representative and diverse sample. The number of students and the geographical location of all educational institutions were obtained from the Ministry of Education [34]. A total of 4864 students were found in the central area and 1220 in the city’s periphery.

The open source calculator Open Epi version 3.01 was used to determine the sample size [35]. A confidence level of 95%, confidence limits as % of 100 (absolute +/−%) of 5%, and prevalence of 26.4% for the center area and 22.2% for the peripheral area were used. The latter values were obtained from the literature on using oral health services in Peruvian children under 12 years of age [20]. The samples for the central and peripheral zones were 282 and 219, respectively.

**Figure 2:**
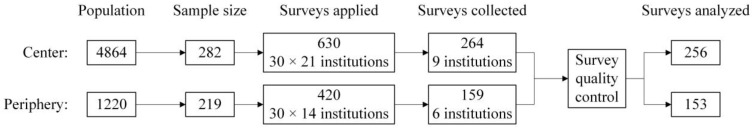
Sample calculation and analyzing surveys.

Invitations to participate in the study were sent to the directors of the city’s 35 primary and initial educational institutions by the Dean of the Facultad de Ciencias de la Salud of the UNTRM. A random sampling technique was used within each institution, where the director defined the classroom or classrooms that would participate in the study. Thirty surveys per educational institution were distributed for application to ensure that the sample was reached (Figure 2), given the population’s previous lack of interest in participating in this type of study [22].

### 2.3. Data Collection and Instrumentation

This study was based on the questionnaire of a previous study [2], to which questions from other studies [3,18,36,37] and of interest to the project were added. The questionnaire covered socioeconomic and demographic information, parental knowledge and attitudes about oral health in primary dentition, and the presence of oral problems in children. For the validation of the survey, the survey was presented to a panel of five judges from the stomatology specialty in the city of Chachapoyas to score it on form (9 criteria) and content (7 criteria) with a Likert-type scale from 1 to 5 [38]. From this score, Cronbach’s alpha test was used in SPSS Statistics 27 [39], obtaining a “high” value of 0.91 [33].

Then, data collection took place between August and September 2023, with a visit to each educational institution. Two members of the research group, in coordination with the classroom teacher, distributed the informed consent protocol and the questionnaire in printed form (Supplementary Materials File S1) to the students, with the instruction to give them to their parents. The protocol outlines the research objective, data processing procedures, and contact information for inquiries, and emphasizes that the questionnaire is voluntary and anonymous. The questionnaire was assigned to the students as a non-mandatory task that their parents could complete within a maximum of two weeks. Subsequently, the students returned the completed questionnaires to their teacher. The inclusion criteria for participation in the study were parents who (a) were able to read and write, (b) provided signed informed consent, and (c) spent more than ten hours per day with the student [22]. The participation of 15 institutions was achieved, with 409 parents correctly completing surveys (256 in the center area and 153 in the city’s peripheral area) (Figure 2).

### 2.4. Ethical Considerations

All respondents gave informed consent before completing the questionnaire. Respondents received complete information about the study and were allowed to ask questions. In addition to the consent form, ethical authorization for the study was obtained from the Facultad de Ciencias de la Salud of the UNTRM.

### 2.5. Data Analysis

Three dependent variables were established, including (i) awareness of the importance of taking the child to the dentist at the primary dentition stage, (ii) knowledge of the harmful effects of children’s habits, and (iii) presence of children’s oral problems. The independent variables were mainly socioeconomic and demographic. Since the independent variables were multiple responses, multinomial logistic regression was used with 95% reliability [40], following the approach of a similar study [41]. SPSS Statistics 27 was used.

## 3. Results and Discussion

### 3.1. Socioeconomic and Demographic Characteristics of Respondents

The majority of respondents were female (75.8%), belonged to the 21–40 years age group (71.4%), and had the Seguro Integral de Salud (SIS), Peruvian public insurance (62.6%) (Table 1). Among the respondents, 49.6% (203) have higher education, and 10.8% (44) are educated in health-related professions. The proportion of parents with higher education is considerably higher in schools in the center (40.3%) than in the city’s periphery (9.3%). The monthly family income for the majority of respondents (72.6%) is below PEN 2000 (<USD 520). There is a higher proportion of families with incomes above USD 520 in schools in the center (23.2%) compared to the city’s periphery (4.2%). Most children are either under 6 years of age (37.7%) or over 10 years of age (33.3%).

### 3.2. Importance of Taking Your Child to the Dentist in the Primary Dentition Stage

**Figure 3:**
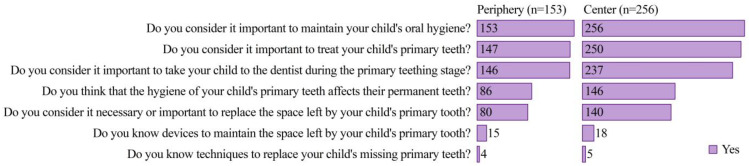
Importance of taking the child to the dentist at the primary dentition stage (*n* = 409).

There was high level of awareness among parents regarding the maintenance of oral hygiene in children (100%) and the importance of both treating primary teeth (97.1%) and taking their child to the dentist at the primary dentition stage (93.6%) (Figure 3). Similar values were observed in previous studies [2,37,43], where most parents were aware of the importance of primary teeth, which could be considered an expected behavior in capital cities of developing countries. Among the respondents, 56.7% were aware that hygiene of the primary teeth also affects the permanent teeth. In contrast, previous studies [36,44,45] observed that most parents did not consider primary teeth important, considering that these are temporary teeth that will fall out and be replaced by permanent teeth, which could be regarded as a cultural belief. Almost 54% of the parents considered it essential to replace the space lost in the primary teeth. However, only a small percentage of parents were familiar with the appliances used to maintain space in the primary dentition (8.1%), and even fewer had knowledge of techniques to replace their child’s missing primary teeth (2.2%).

Female respondents were more aware of caring for their child’s dental health (odd ratio, OR = 0.484; *p* = 0.037) (Table 2). Similar findings were reported by previous studies [17,46,47] and could be attributed to the fact that, in general, the child’s primary caregiver is the mother, compared to the father, who is more involved in financial support [48]. Respondent parents with higher educational levels are twice as aware of the importance of taking their child to the dentist at the primary teeth stage (OR = 2.144, *p* = 0.043) (Table 2). It is known that the level of education parents possess and their employment status can influence their children’s oral health [10,17]. In families with children in the early teething stage, parents with higher educational levels tend to have better oral health knowledge [49]. Insurance such as EsSalud leads to parents being three times more aware of taking their child to the dentist than those without health insurance (OR = 2.627, *p* = 0.044) (Table 2). However, in previous studies in the population of children affiliated with this social insurance (EsSalud), the prevalence of dental caries was 79.8% in children aged 3 to 5 years and 90.4% in those aged 12 years [20,50].

### 3.3. Knowledge about Children’s Habits and Their Prejudicial Effects

**Figure 4:**
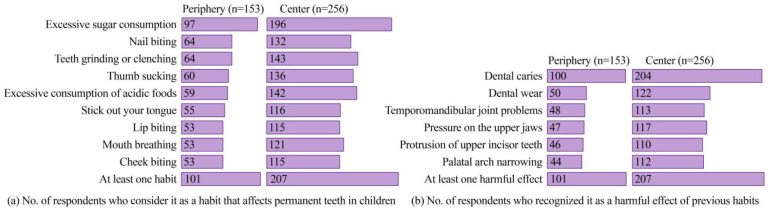
Knowledge about children’s habits and their harmful effects (*n* = 409).

Among the parents surveyed, 75.3% (308/409) identify at least one habit affecting permanent teeth and at least one detrimental effect of these habits (Figure 4). The proportion of parents with this knowledge is higher in educational institutions in the central area (80.9% = 207/256) compared to those in the periphery (66.0% = 101/153) of the city. Excessive sugar consumption is the most widely known harmful habit (71.6%, 293 of 409 respondents), while the recognition of other habits is less than 50.6% (Figure 4a). This confirms the findings of previous studies [9,10] that determined that dietary intake of sweets, fast foods, and sugar-sweetened beverages increased the risk of severe early childhood caries. Dental caries is the most recognized detrimental effect (74.3%, 304 of 409 respondents), while the other effects are recognized by less than 42.1% (Figure 4b). This is because dental caries affects between 60% and 90% of school-aged [5] and preschool-aged [15] children, respectively. In addition, it is one of the most frequent causes of premature loss of baby teeth [4]. The pattern of proportions of habits and the best-known effects is similar in both respondents from educational institutions in the peripheral area and the city’s center (Figure 4).

Parents whose children study in educational institutions in the city center are twice as likely to recognize dental caries as a harmful effect of their children’s bad habits (OR = 2.079, *p* = 0.001). On the other hand, if the monthly household income is between PEN 2000 and 3000 (approx. USD 520–780), there is a greater chance that parents will identify a dental caries problem (*p* = 0.028; OR = 3.504) (Table 3). This suggests that determinants, such as the level of monthly household income, could explain the high prevalence rates of dental caries [12]. Additionally, parents whose children study in inner-city educational institutions are twice as likely to recognize palatal arch narrowing (OR = 1.927, *p* = 0.003), protrusion of the upper incisor teeth (OR = 1.753, *p* = 0.010), tooth wear (OR = 1.876, *p* = 0.003), temporomandibular joint problems (OR = 1.729, *p* = 0.011), and pressure on the jaws (OR = 1.898, *p* = 0.003) as harmful effects of children’s bad habits. This could be related to the parent’s educational level, considering that parents with higher academic levels tend to have more oral care needs, such as pit and fissure sealants, and even their children tend to perform better oral hygiene practices [49].

### 3.4. Presence of Dental Problems in Children

**Figure 5:**
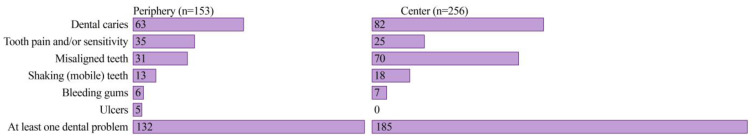
Presence of dental problems in the children (*n* = 409).

Among the parents surveyed, 77.5% (317/409) report that their child presents at least one dental problem; the proportion is higher in educational institutions located in the peripheral area (86.3% = 132/153) compared to those in the center (72.3% = 185/256) of the city (Figure 5). Children who study in educational institutions in the city center present a protective factor for the possibility of introducing a dental problem (*p* = 0.001; OR = 0.415) (Table 4). Those who reported having a monthly family income of less than PEN 1000 (approx. USD 260) present a higher risk of their children having dental problems (*p* = 0.002; OR = 4.775). This may explain that monthly family income may modify the association between the high frequency of dental caries and sugar intake [51]. Additionally, the highest prevalence of untreated dental caries is concentrated among the most socioeconomically disadvantaged children [52].

The three moments in which the respondent takes their child to the dentist represent a risk factor; however, if they take their child after the eruption of all their permanent teeth, the risk of suffering a dental problem is much higher (*p* = 0.000; OR = 11.61) (Table 4). Not examining your child’s teeth from the first year of life represents an increased risk of your child having a dental problem (*p* = 0.046; OR = 1.610). New models of managing caries prevention in children at an earlier age call for children to first visit the dentist at the age of 1 year or when their first tooth erupts [53]. If the frequency of brushing is once a day, there will be a seven times greater chance that your child will have a dental problem (OR = 6.786; *p* = 0.015). A higher risk factor for dental problems was observed in children aged 8 to 10 years (*p* = 0.004; OR = 3.280). The fact that the parents had a high level of education was a protective factor for their child not having a dental problem (*p* = 0.001; OR = 0.361), as was having insurance such as EsSalud (*p* = 0.000; OR = 0.318). This is related to previous findings [49] that identified that children from families with an excellent educational level were more likely to brush their teeth, do so more often, visit the dentist more frequently, and undergo regular dental checkups.

#### 3.4.1. Presence of Pain and Sensitivity of Teeth in the Children

Children who attend educational institutions in the city center are less likely to suffer from toothache and sensitivity (*p* = 0.000; OR = 0.365) (Table 5). This result coincides with previous research indicating that socioeconomic factors affect children’s oral health, with more unfavorable indicators in marginal areas [54,55]. Parents who do not consider nail biting as a harmful habit increase the risk of their children experiencing future problems such as tooth pain and sensitivity (*p* = 0.023; OR = 0.175). It is important to note that poor oral habits, including nail biting, can have adverse effects on the development of hard tissues and overall dental hygiene [56,57]. In addition, it was shown that parents who never take their children to the dentist are the ones who presented the highest risk factor for their children to have dental pain or sensitivity (*p* = 0.006; OR = 1.125). This attitude may be due to parental fear or anxiety [58].

#### 3.4.2. Presence of Dental Malocclusion in Children

In our study, the peripheral zone (20.3%) had fewer parents reporting dental malocclusion compared to the central zone (27.3%). A previous study conducted in three Peruvian cities reported the same pattern [59]. Children whose parents do not consider excess sugar consumption a bad habit (Figure 4a) are at a higher risk of developing dental malocclusion (*p* = 0.005; OR = 0.377) (Table 6), as reported in previous studies [54,58]. Parents’ lack of knowledge about dental caries as a harmful effect (Figure 4b) represents a risk factor for dental malocclusion in their children (*p* = 0.005; OR = 0.388). Specifically, parents are unaware due to low educational levels [60]. Parents who wait for their child to present tooth pain before taking them to a dentist increase the risk of misaligned teeth (*p* = 0.027; OR = 1.871). In this sense, initiating dental checkups from an early age reduces the risks of dental malocclusion [61]. In addition, children whose teeth were not examined from the first year of life will have a 2.3 times greater chance of presenting problems with misaligned teeth (*p* = 0.001).

#### 3.4.3. Presence of Mobile Teeth in the Children

A recognition by parents of the possible harmful effects of certain habits on their children’s jaws is crucial for preventing oral health problems [62]. In our study, parents who are not able to recognize that pressure on the jaws is a harmful effect (Figure 4b) of certain habits of their children such as thumb sucking (Figure 4a) constitute a risk of their children having mobile teeth (*p* = 0.046; OR = 1.177) (Table 7). This finding highlights the need for parents to be attentive to signs of excessive pressure on their children’s teeth, such as lip/cheek pulling or finger biting, which could be linked to an increased risk of loose teeth.

#### 3.4.4. Presence of Dental Caries in Children

Children from households with an income below PEN 1000 face a higher risk of experiencing dental caries problems (*p* = 0.012; OR = 13.636) (Table 8). An association has been found between low socioeconomic status and worsening child oral health [55,63]. Children whose parents are unable to recognize problems such as palatal arch narrowing in their children are 13 times more likely to develop caries problems (*p* = 0.015). In fact, ignorance of dental anomalies is linked to worse oral health [56]. Parents who do not recognize temporomandibular problems in their children are putting them at risk of developing caries (*p* = 0.021; OR = 0.131). Parents who postpone seeking dental care for their children until they experience toothaches (*p* = 0.000; OR = 2.703) or who neglect to take them to the dentist’s office (*p* = 0.040; OR = 2.299) significantly heighten their children’s risk of developing caries. This type of behavior from parents, characterized by a low frequency of dental check-ups and treatments, is linked to the deterioration of oral health [61,64].

### 3.5. Strengths, Limitations, and Implications for Future Research

Although the validity of the questionnaire was tested by experts, more robust results could be obtained by validating the surveys with a test group of parents. New strategies must also be sought to reach a greater number of participants. However, these first results are useful for implementing and evaluating educational programs aimed at improving parents’ knowledge of oral health in the primary dentition stage, especially in the peripheral areas of the city, which are the most vulnerable. The results can be generalized with caution to the high Andean cities of the country, with similar sociodemographic characteristics. The cross-sectional design only allows us to infer associations and not causality. Future longitudinal studies would allow us to evaluate the evolution of knowledge and its impact on behaviors and oral health in the long term. Parents’ oral problems and health-seeking behaviors and their relationship to their children’s dental health should also be analyzed.

## 4. Conclusions

The knowledge and attitudes of parents regarding oral health during the primary dentition stage were assessed in the high Andean city of Chachapoyas, Amazonas department, northern Peru. Responses were collected from a total of 409 parents (256 in the center and 153 in the periphery) from 15 educational institutions. The results showed a good general awareness of the importance of oral hygiene and attending the dentist during the primary dentition stage. However, there are gaps in knowledge regarding techniques to prevent tooth space loss. Geographic and socioeconomic factors such as educational level and family income significantly influence knowledge and the presence of oral problems in children. In addition, some parents do not fully recognize the harmful effects of bad habits such as excessive sugar consumption. This exposes them to a higher risk of presenting problems such as caries, dental malocclusion, or mobile teeth. There is a need to strengthen oral health education for parents, especially in peripheral and lower socioeconomic status areas of the cities, to improve the prevention of dental disorders in early childhood. This research contributes to the scientific literature and provides relevant information for the design of effective interventions to promote better oral health in children in this city.

## Figures and Tables

**Table 1:** Socioeconomic/demographic characteristics of respondents and age of their children.

Variable		The Area Where the Child’s School Is Located		Total *N* = 409 (100%)
		Periphery *n* (%) *n* = 153 (37.4%)	Center *n* (%) *n* = 256 (62.6%)	
Respondent’s sex	Female	124 (30.3%)	186 (45.5%)	310 (75.8%)
	Male	29 (7.1%)	70 (17.1%)	99 (24.2%)
Respondent’s age (years)	Below 20	6 (1.5%)	0 (0%)	6 (1.5%)
	21 to 40	115 (28.1%)	177 (43.3%)	292 (71.4%)
	41 to 60	29 (7.1%)	75 (18.3%)	104 (25.4%)
	Above 61	3 (0.7%)	4 (1%)	7 (1.7%)
Respondent’s health insurance ^1^	EsSalud	21 (5.1%)	107 (26.2%)	128 (31.3%)
	SIS	125 (30.6%)	131 (32%)	256 (62.6%)
	None	7 (1.7%)	18 (4.4%)	25 (6.1%)
Respondent’s highest educational level	Primary school	46 (11.2%)	24 (5.9%)	70 (17.1%)
	Secondary school	53 (13%)	57 (13.9%)	110 (26.9%)
	Higher education	38 (9.3%)	165 (40.3%)	203 (49.6%)
	None	16 (3.9%)	10 (2.4%)	26 (6.4%)
Health-related higher education?	Yes	10 (2.4%)	34 (8.3%)	44 (10.8%)
	No	28 (6.8%)	131 (32%)	159 (38.9%)
Monthly family income in Peruvian soles (Peruvian soles = 0.27 USD)	Less than 1000	95 (23.2%)	79 (19.3%)	174 (42.5%)
	1000–2000	41 (10%)	82 (20%)	123 (30.1%)
	2000–3000	10 (2.4%)	54 (13.2%)	64 (15.6%)
	3000–5000	1 (0.2%)	28 (6.8%)	29 (7.1%)
	More than 5000	6 (1.5%)	13 (3.2%)	19 (4.6%)
Child’s age (years)	Below 6	56 (13.7%)	98 (24%)	154 (37.7%)
	6 to 8	24 (5.9%)	34 (8.3%)	58 (14.2%)
	8 to 10	37 (9%)	24 (5.9%)	61 (14.9%)
	Above 10	36 (8.8%)	100 (24.4%)	136 (33.3%)

^1^ The Peruvian health system has two main sectors, public and private. In the public sector, there is the Seguro Integral de Salud (SIS) with a subsidized regime, and Health Social Security (EsSalud) with an essentially contributory regime. The SIS is a government program for the uninsured population, with services provided through the network of facilities of the Ministry of Health. EsSalud is the social insurance for the salaried population and their families, with services in its facilities, although it can also purchase services from the private sector [42].

**Table 2:** Multinomial logistic regression on the importance of taking the child to the dentist at the primary dentition stage ^1^.

Independent Variables		Regression Coefficient (B)	Exponential of B/Odd Ratio	95% Confidence Interval (Lower–Upper Limits)	*p*-Value
Respondent’s sex	Female	−0.725	0.484	1.212–2.205	0.037
	Male	0 ^2^			
Respondent’s highest educational level	Higher education	0.763	2.144	1.880–5.222	0.043
	None	1.022	2.778	0.342–22.539	0.339
	Primary school	0.606	1.833	0.560–6.002	0.316
	Secondary school	0 ^2^			
Respondent’s health insurance	EsSalud	0.966	2.627	1.879–7.855	0.044
	None	−0.026	0.975	0.214–4.435	0.973
	SIS	0 ^2^			

^1^ The reference category is: No. ^2^ This parameter has been set to zero because it is redundant.

**Table 3:** Multinomial logistic regression on knowledge of harmful effects of children’s habits.

Dependent Variable ^1^ (Figure 4b)	Independent Variable		Regression Coefficient (B)	Exponential of B/Odd Ratio	95% Confidence Interval (Lower–Upper Limits)	*p*-Value
Dental caries	The area where the child’s school is located	Center	0.732	2.079	1.324–3.265	0.001
		Periphery	0 ^2^			
	Monthly family income in Peruvian soles	Less than 1000	0.507	1.660	0.632–4.364	0.304
		1000–2000	0.903	2.468	0.905–6.731	0.078
		2000–3000	1.254	3.504	1.145–10.724	0.028
		3000–5000	1.250	3.491	0.927–13.144	0.065
		More than 5000	0 ^2^			
Palatal arch narrowing	The area where the child’s school is located	Center	0.656	1.927	1.255–2.957	0.003
		Periphery	0 ^2^			
Protrusion of upper incisor teeth	The area where the child’s school is located	Center	0.561	1.753	1.146–2.681	0.010
		Periphery	0 ^2^			
Dental wear	The area where the child’s school is located	Center	0.629	1.876	1.235–2.847	0.003
		Periphery	0 ^2^			
Temporomandibular joint problems	The area where the child’s school is located	Center	0.547	1.729	1.134–2.634	0.011
		Periphery	0 ^2^			
Pressure on the upper jaws	The area where the child’s school is located	Center	0.641	1.898	1.244–2.896	0.003
		Periphery	0 ^2^			

^1^ The reference category is: No. ^2.^ This parameter has been set to zero because it is redundant.

**Table 4:** Multinomial logistic regression on the presence of any dental problem in the children ^1^.

Independent Variables		Regression Coefficient (B)	Exponential of B/Odd Ratio	95% Confidence Interval (Lower–Upper Limits)	*p*-Value
The area where the child’s school is located	Center	−0.881	0.415	0.243–0.708	0.001
	Periphery	0 ^2^			
Monthly family income in Peruvian soles	Less than 1000	1.563	4.775	1.738–13.121	0.002
	1000–2000	1.048	2.851	1.037–7.836	0.042
	2000–3000	0.260	1.296	0.456–3.683	0.626
	3000–5000	−0.111	0.895	0.278–2.879	0.853
	More than 5000	0 ^2^			
When do you take your child to the dentist?	After the eruption of all permanent teeth	2.452	11.611	3.428–39.331	0.000
	Never	1.342	3.826	1.487–9.847	0.005
	When the child has tooth pain	1.656	5.237	3.054–8.981	0.000
	Without the need for oral treatment	0 ^2^			
Do you examine your child’s teeth from the first year of life?	No	0.476	1.610	1.008–2.573	0.046
	Yes	0 ^2^			
How many times a day does your child brush their teeth?	1	1.915	6.786	1.451–31.724	0.015
	2	0.762	2.143	0.633–7.256	0.221
	3	0.943	2.568	0.780–8.462	0.121
	More than 3	0.437	1.548	0.345–6.942	0.568
	Not applicable	0 ^2^			
Student age range (years)	6 to 8	0.442	1.556	0.782–3.099	0.208
	8 to 10	1.188	3.280	1.451–7.416	0.004
	Above 10	1.115	3.049	1.691–5.498	0.000
	Below 6	0 ^2^			
Respondent’s highest educational level	Higher education	−1.018	0.361	0.197–0.663	0.001
	None	−0.066	0.936	0.285–3.077	0.913
	Primary school	0.427	1.532	0.596–3.937	0.376
	Secondary school	0 ^2^			
Respondent’s health insurance	EsSalud	−1.146	0.318	0.195–0.519	0.000
	None	0.335	1.398	0.400–4.889	0.599
	SIS	0 ^2^			

^1^ The reference category is: No. ^2^ This parameter has been set to zero because it is redundant.

**Table 5:** Multinomial logistic regression on tooth pain and sensitivity in children ^1^.

Independent Variables		Regression Coefficient (B)	Exponential of B/Odd Ratio	95% Confidence Interval (Lower–Upper Limits)	*p*-Value
The area where the child’s school is located	Center	−1.008	0.365	0.209–0.638	0.000
	Periphery	0 ^2^			
Does the child bite his nails?	No	−1.744	0.175	0.039–0.789	0.023
	Yes	0 ^2^			
When do you take your child to the dentist?	After the eruption of all permanent teeth	1.436	4.205	1.375–12.864	0.012
	Never	1.634	5.125	1.598–16.432	0.006
	When the child has tooth pain	1.615	5.028	2.063–12.259	0.000
	Without the need for oral treatment	0 ^2^			

^1^ The reference category is: No. ^2^ This parameter has been set to zero because it is redundant.

**Table 6:** Multinomial logistic regression on the presence of dental malocclusion in children ^1^.

Independent Variables		Regression Coefficient (B)	Exponential of B/Odd Ratio	95% Confidence Interval (Lower–Upper Limits)	*p*-Value
Do you consider excessive sugar consumption as a habit that affects permanent teeth in children?	No	−0.976	0.377	0.189–0.750	0.005
	Yes	0 ^2^			
Do you recognize dental caries as a harmful effect of the habits in Figure 4a?	No	−0.945	0.388	0.201–0.750	0.005
	Yes	0 ^2^			
When do you take your child to the dentist?	After the eruption of all permanent teeth	0.724	2.063	0.950–4.481	0.067
	Never	0.665	1.945	0.819–4.621	0.132
	When the child has tooth pain	0.626	1.871	1.075–3.256	0.027
	Without the need for oral treatment	0 ^2^			
Do you examine your child’s teeth from the first year of life?	No	0.851	2.341	1.413–3.879	0.001
	Yes	0 ^2^			

^1^ The reference category is: No. ^2^ This parameter has been set to zero because it is redundant.

**Table 7:** Multinomial logistic regression on the presence of mobile teeth in children ^1^.

Independent Variables		Regression Coefficient (B)	Exponential of B/Odd Ratio	95% Confidence Interval (Lower–Upper Limits)	*p*-Value
Do you recognize pressure on the upper jaws as a detrimental effect of the habits in Figure 4a?	No	−1.731	1.177	1.030–1.247	0.046
	Yes	0 ^2^			

^1^ The reference category is: No. ^2^ This parameter has been set to zero because it is redundant.

**Table 8:** Multinomial logistic regression on the presence of dental caries in children ^1^.

Independent Variables		Regression Coefficient (B)	Exponential of B/Odd Ratio	95% Confidence Interval (Lower–Upper Limits)	*p*-Value
Monthly family income in Peruvian soles	Less than 1000	2.613	13.636	1.780–104.443	0.012
	1000–2000	2.340	10.385	1.341–80.411	0.025
	2000–3000	2.174	8.791	1.098–70.377	0.041
	3000–5000	0.731	2.077	0.200–21.596	0.541
	More than 5000	0 ^2^			
Do you recognize palatal arch narrowing as a detrimental effect of the habits in Figure 4a?	No	2.585	13.264	1.642–107.152	0.015
	Yes	0 ^2^			
Do you recognize temporomandibular joint problems as a detrimental effect of the habits in Figure 4a?	No	−2.033	0.131	0.023–0.740	0.021
	Yes	0 ^2^			
When do you take your child to the dentist?	After the eruption of all permanent teeth	0.480	1.616	0.771–3.387	0.203
	Never	0.832	2.299	1.041–5.078	0.040
	When the child has tooth pain	0.994	2.703	1.640–4.454	0.000
	Without the need for oral treatment	0 ^2^			

^1^ The reference category is: No. ^2^ This parameter has been set to zero because it is redundant.

## Data Availability

The data used to support the findings of this study are available from the corresponding author upon request.

## Supplementary Materials

The following supporting information can be downloaded at: https://www.mdpi.com/article/10.3390/ijerph21020154/s1, File S1: Informed consent protocol and the questionnaire.

## References

[1] Nadelman P., Magno M.B., Pithon M.M., de Castro A.C.R., Maia L.C. (2021). Does the Premature Loss of Primary Anterior Teeth Cause Morphological, Functional and Psychosocial Consequences?. Braz. Oral Res..

[2] Ramakrishnan M., Banu S., Ningthoujam S., Samuel V. (2019). Evaluation of Knowledge and Attitude of Parents about the Importance of Maintaining Primary Dentition—A Cross-Sectional Study. J. Fam. Med. Prim Care.

[3] Vittoba S.J., Srinivasan I. (2016). Knowledge and Awareness of Primary Teeth and Their Importance among Parents in Bengaluru City, India. Int. J. Clin. Pediatr. Dent..

[4] Alqarni A.S., AlGomaiah M.A. (2023). Evaluation of the Efficacy of Various Root Canal Irrigants on Removal of Smear Layer in Primary Teeth: A Comparative Study. J. Pharm. Bioallied Sci..

[5] Iheozor-Ejiofor Z., Worthington H.V., Walsh T., O’Malley L., Clarkson J.E., Macey R., Alam R., Tugwell P., Welch V., Glenny A.-M. (2015). Water Fluoridation for the Prevention of Dental Caries. Cochrane Database Syst. Rev..

[6] Organización Mundial de la Salud (2022). Informe Sobre La Situación Mundial de La Salud Bucodental: Hacia La Cobertura Sanitaria Universal Para Salud Bucodental de Aquí a 2030. Resumen Ejecutivo.

[7] Xu N., Deng S., Liang Y., Chen A., Zou D., Li L., Qiu R. (2023). Impact of Migration on Oral Health Outcomes of Children in Multi-Beneficial Kindergartens in Nanning, Southern China: A Cross-Sectional Study. BMC Oral Health.

[8] Perera P.J., Fernando M.P., Warnakulasooriya T.D., Ranathunga N. (2014). Effect of Feeding Practices on Dental Caries among Preschool Children: A Hospital Based Analytical Cross Sectional Study. Asia Pac. J. Clin. Nutr..

[9] Han D., Kim D., Kim M., Kim J., Jung-Choi K., Bae K. (2014). Regular Dental Checkup and Snack–Soda Drink Consumption of Preschool Children Are Associated with Early Childhood Caries in Korean Caregiver/Preschool Children Dyads. Community Dent. Oral Epidemiol..

[10] Schroth R.J., Halchuk S., Star L. (2013). Prevalence and Risk Factors of Caregiver Reported Severe Early Childhood Caries in Manitoba First Nations Children: Results from the RHS Phase 2 (2008–2010). Int. J. Circumpolar Health.

[11] Chankanka O., Cavanaugh J.E., Levy S.M., Marshall T.A., Warren J.J., Broffitt B., Kolker J.L. (2011). Longitudinal Associations between Children’s Dental Caries and Risk Factors. J. Public Health Dent..

[12] Pinto-Sarmento T.C.D.A., Abreu M.H., Gomes M.C., Costa E.M.M.D.B., Martins C.C., Granville-Garcia A.F., Paiva S.M. (2016). Determinant Factors of Untreated Dental Caries and Lesion Activity in Preschool Children Using ICDAS. PLoS ONE.

[13] Dye B.A., Vargas C.M., Lee J.J., Magder L., Tinanoff N. (2011). Assessing the Relationship Between Children’s Oral Health Status and That of Their Mothers. J. Am. Dent. Assoc..

[14] Wigen T.I., Espelid I., Skaare A.B., Wang N.J. (2011). Family Characteristics and Caries Experience in Preschool Children. A Longitudinal Study from Pregnancy to 5 Years of Age. Community Dent. Oral Epidemiol..

[15] Zeng L., Zeng Y., Zhou Y., Wen J., Wan L., Ou X., Zhou X. (2018). Diet and Lifestyle Habits Associated with Caries in Deciduous Teeth among 3- to 5-Year-Old Preschool Children in Jiangxi Province, China. BMC Oral Health.

[16] Moulana S., Yashoda R., Puranik M., SHiremath S., Rahul G. (2012). Knowledge, Attitude and Practices towards Primary Dentition among the Mothers of 3–5 Year Old Pre-School Children in Bangalore City. J. Indian Assoc. Public Health Dent..

[17] Ansari S., Alanazi A., Alqahtani M., Alharbi A., Hodan F., Alshaye R. (2020). Perception of Saudi Parents towards the Problems Related to Primary Dentition of Their Children Residing in Riyadh City. J. Fam. Med. Prim. Care.

[18] Manohar J., Mani G. (2017). Knowledge and Attitude of Parents Regarding Children’s Primary Teeth & Their Willingness for Treatment. J. Pharm. Sci. Res..

[19] Setty J., Srinivasan I. (2011). Awareness and Attitude of Patients′ Parents toward Pulp Therapy of the Primary Teeth: A Clinical Survey. J. Indian Soc. Pedod. Prev. Dent..

[20] Azañedo D., Hernández-Vásquez A., Visconti-Lopez F.J., Turpo Cayo E.Y. (2023). Frequency, Inequalities and Spatial Distribution of Oral Health Services Utilization in Peruvian Children under Twelve Years of Age: A Population-Based Comparative Analysis of the Years 2017 and 2021. BMC Oral Health.

[21] Chávez J.D. (2018). Nivel de Conocimiento Sobre Salud Bucal En Los Padres de Los Alumnos Con Síndrome de Down. Bachelors’s Thesis.

[22] Victorio-Pérez J., Mormontoy-Laurel W., Díaz-Pizán M.E. (2019). Conocimientos, Actitudes y Prácticas de Padres/ Cuidadores Sobre Salud Bucal En El Distrito de Ventanilla. Rev. Estomatológica Hered..

[23] Cabrera C.C.B. (2022). Relación Entre Nivel de Conocimiento Sobre Salud Bucal y Hábitos Orales Nocivos En Padres de Familia de Alumnos de 1er., 2do. y 3er. Grado de Educación Primaria de La IE. San Pedro, Distrito de Chimbote, Provincia Del Santa, Departamento de Áncash, Año 2020. Bachelors’s Thesis.

[24] Vásquez M.K. (2017). Nivel de Conocimiento En Salud Bucal de Padres de Familia de La Institución Educativa 11011 “Señor de Los Milagros” Del Distrito de José Leonardo Ortiz, 2016. Bachelors’s Thesis.

[25] Clavijo V.A.J., Campos C.K. (2023). Nivel de Conocimiento Sobre Salud Oral de Los Padres y Su Relación Con Hábitos de Higiene Oral de Sus Hijos de 8 a 10 Años. Odontol Pediatr.

[26] Sánchez H.F.M., Kanashiro I.C. (2022). Conocimientos, Actitudes y Prácticas de Los Padres Respecto a La Salud Bucal de Sus Hijos En El Hospital Edgardo Rebagliati Martins, En Tiempos de Pandemia COVID-19. Odontol. Pediatr..

[27] Roque T.L.A., Tello Q.S.M. (2020). Nivel de Conocimiento y Conducta Sobre Salud Bucal En Padres de Una Institución Educativa de Nivel Inicial y Primaria, Pasco2021. Bachelors’s Thesis.

[28] Díaz A.S.M. (2022). Nivel de Conocimiento Sobre Salud Oral En Padres de Niños En Edad Preescolar de Un Centro de Salud, Arequipa 2022. Bachelors’s Thesis.

[29] Suarez S.G.G., Carhuas C.D.D. (2022). Conocimiento y Actitud Sobre Salud Oral En Padres de Los Niños Atendidos En El Hospital de Apoyo Jesús Nazareno, Ayacucho—2022. Bachelors’s Thesis.

[30] Silva-López J.O., Salas López R., Rojas Briceño N.B., Gómez Fernández D., Terrones Murga R.E., Iliquín Trigoso D., Barboza Castillo E., Oliva Cruz M., Barrena Gurbillón M.Á. (2022). Analytic Hierarchy Process (AHP) for a Landfill Site Selection in Chachapoyas and Huancas (NW Peru): Modeling in a GIS-RS Environment. Adv. Civ. Eng..

[31] INEI (2018). Amazonas. Resultados Definitivos de Censos Nacionales 2017: XII de Población, VII de Vivienda y III de Comunidades Indígenas.

[32] INEI (2022). Perú: Proyecciones de Población Total Según Departamento, Provincia y Distrito, 2018-2022.

[33] Hernández S.R., Fernández C.C., Baptista L.M.P. (2014). Metodología de La Investigación.

[34] MINEDU ESCALE—Unidad de Estadística Educativa. https://sigmed.minedu.gob.pe/mapaeducativo/.

[35] Dean A., Sullivan K., Soe M. OpenEpi: Open Source Epidemiologic Statistics for Public Health, Versión 3.01. www.OpenEpi.com.

[36] Vadakkepurayil K., Balakrishnan B.A. (2023). Knowledge and Awareness About Importance of Primary Dentition Among Parents and Pediatricians. J. South Asian Assoc. Pediatr. Dent..

[37] Chandran V., Varma R.B., Joy T., Ramanarayanan V., Govinda B., Menon M. (2019). Parental Knowledge, Attitude, and Practice Regarding the Importance of Primary Dentition of Their Children in Kerala, India. J. Indian Assoc. Public Health Dent..

[38] López N.D.L., Pérez-Almagro M.-C., Caro-Rivas M.-A. (2024). Aplicación Del Método Delphi Para La Validación de Un Instrumento Para Medir Actitudes, Conocimientos y Uso de Estrategias Pedagógicas Interdisciplinares. REIRE Rev. D’innovació I Recer. En Educ..

[39] Rodríguez-Rodríguez J., Reguant-Álvarez M. (2020). Calcular La Fiabilitat d’un Qüestionari o Escala Mitjançant l’SPSS: El Coeficient Alfa de Cronbach. REIRE Rev. D’innovació I Recer. En Educ..

[40] Blanco-Reina E., Valdellós J., Ocaña-Riola R., García-Merino M.R., Aguilar-Cano L., Ariza-Zafra G., Bellido-Estévez I. (2019). Factors Associated with Health-Related Quality of Life in Community-Dwelling Older Adults: A Multinomial Logistic Analysis. J. Clin. Med..

[41] Uguru N., Onwujekwe O., Uguru C., Ogu U., Okwuosa C., Okeke C. (2021). Oral Health-Seeking Behavior among Different Population Groups in Enugu Nigeria. PLoS ONE.

[42] Mezones-Holguin E., Amaya E., Bellido-Boza L., Mougenot B., Murillo J.P., Villegas-Ortega J., Del-Carmen J. (2019). Cobertura de Aseguramiento En Salud: El Caso Peruano Desde La Ley de Aseguramiento Universal. Rev. Peru Med. Exp. Salud Publica.

[43] Thakare V.G., Ajith Krishnan C.G., Chaware S. (2012). Parents′ Perceptions of Factors Influencing the Oral Health of Their Preschool Children in Vadodara City, Gujarat: A Descriptive Study. Eur. J. Gen. Dent..

[44] Wong D., Perez-Spiess S., Julliard K. (2005). Attitudes of Chinese Parents toward the Oral Health of Their Children with Caries: A Qualitative Study. Pediatr. Dent..

[45] Nagaveni N.B., Radhika N.B., Umashankar K.V. (2011). Knowledge, Attitude and Practices of Parents Regarding Primary Teeth Care of Their Children in Davangere City, India. Pesqui Bras. Odontopediatria Clin. Integr..

[46] Chhabra N., Chhabra A. (2012). Parental Knowledge, Attitudes and Cultural Beliefs Regarding Oral Health and Dental Care of Preschool Children in an Indian Population: A Quantitative Study. Eur. Arch. Paediatr. Dent..

[47] Rajab L.D., Petersen P.E., Bakaeen G., Hamdan M.A. (2002). Oral Health Behaviour of Schoolchildren and Parents in Jordan. Int. J. Paediatr. Dent..

[48] Si Han Y., Pei Jun W. (2013). Parental Involvement in Child’s Development: Father vs. Mother. Open J. Med. Psychol..

[49] Chen L., Hong J., Xiong D., Zhang L., Li Y., Huang S., Hua F. (2020). Are Parents’ Education Levels Associated with Either Their Oral Health Knowledge or Their Children’s Oral Health Behaviors? A Survey of 8446 Families in Wuhan. BMC Oral Health.

[50] Ortiz-León F.A. (2014). Perfil Epidemiológico de Salud Bucal En Niños Atendidos En El Seguro Social Del Perú. Odontol. Pediatr..

[51] Lima L.J.S., da Consolação Soares M.E., Moreira L.V., Ramos-Jorge J., Ramos-Jorge M.L., Marques L.S., Fernandes I.B. (2023). Family Income Modifies the Association between Frequent Sugar Intake and Dental Caries. Int. J. Paediatr. Dent..

[52] Karam S.A., Costa F.D.S., Peres K.G., Peres M.A., Barros F.C., Bertoldi A.D., Santos I.S., Tovo L., Matijasevich A., Menezes A.M.B. (2023). Two Decades of Socioeconomic Inequalities in the Prevalence of Untreated Dental Caries in Early Childhood: Results from Three Birth Cohorts in Southern Brazil. Community Dent. Oral Epidemiol..

[53] Ramos-Gomez F., Crystal Y.O., Ng M.W., Tinanoff N., Featherstone J.D. (2010). Caries Risk Assessment, Prevention, and Management in Pediatric Dental Care. Gen. Dent..

[54] Karamehmedovic E., Bajric E., Virtanen J.I. (2021). Oral Health Behaviour of Nine-Year-Old Children and Their Parents in Sarajevo. Int. J. Environ. Res. Public Health.

[55] Silva-Oliveira F., Goursand D., Ferreira R.C., Paiva P.C.P., Paiva H.N., Ferreira E.F., Zarzar P.M. (2018). Traumatic Dental Injuries in Brazilian Children and Oral Health-related Quality of Life. Dent. Traumatol..

[56] Baghchechi M., Pelletier J.L., Jacob S.E. (2021). Art of Prevention: The Importance of Tackling the Nail Biting Habit. Int. J. Womens Dermatol..

[57] Suligowska K., Mikietyńska M., Pakalska-Korcala A., Wolańczyk T., Prośba-Mackiewicz M., Zdrojewski T. (2021). Parafunctions, Signs and Symptoms of Temporomandibular Disorders (TMD), in Children with Attention-Deficit Hyperactivity Disorder (ADHD). Psychiatr. Pol..

[58] Dabawala S., Suprabha B.S., Shenoy R., Rao A., Shah N. (2017). Parenting Style and Oral Health Practices in Early Childhood Caries: A Case–Control Study. Int. J. Paediatr. Dent..

[59] de Llano-Pérula M.C., Ricse E., Fieuws S., Willems G., Orellana-Valvekens M.F. (2020). Malocclusion, Dental Caries and Oral Health-Related Quality of Life: A Comparison between Adolescent School Children in Urban and Rural Regions in Peru. Int. J. Environ. Res. Public Health.

[60] Mishra A., Pandey R., Chopra H., Arora V. (2018). Oral Health Awareness in School-Going Children and Its Significance to Parent’s Education Level. J. Indian Soc. Pedod. Prev. Dent..

[61] Aburahma S.K., Mhanna A., Al-Mousa S., Al-Nusair J., Al Habashneh R. (2021). Dental Health Status and Hygiene in Children with Cerebral Palsy: A Matched Case-control Study. Int. J. Paediatr. Dent..

[62] Alves C.L., Fagundes D.M., Soares P.B.F., Ferreira M.C. (2019). Knowledge of Parents/Caregivers about Bruxism in Children Treated at the Pediatric Dentistry Clinic. Sleep Sci..

[63] Sfreddo C.S., Moreira C.H.C., Nicolau B., Ortiz F.R., Ardenghi T.M. (2019). Socioeconomic Inequalities in Oral Health-Related Quality of Life in Adolescents: A Cohort Study. Qual. Life Res..

[64] Opydo-Szymaczek J., Borysewicz-Lewicka M., Andrysiak K., Witkowska Z., Hoffmann-Przybylska A., Przybylski P., Walicka E., Gerreth K. (2021). Clinical Consequences of Dental Caries, Parents’ Perception of Child’s Oral Health and Attitudes towards Dental Visits in a Population of 7-Year-Old Children. Int. J. Environ. Res. Public Health.

