# Supplementary material for: Knowledge and Attitudes of Parents about Oral Health in the Primary Dentition Stage in a Peruvian High Andean City
Source: Int J Environ Res Public Health. 2024 Jan 30;21(2):154. doi: 10.3390/ijerph21020154 (PMC10888232; doi:10.3390/ijerph21020154)
Supplement: Supplementary file 1 [file ijerph-21-00154-s001.zip › ijerph-2761855-Supplementary Material.pdf]

Protocolo de consentimiento informado para encuestas
Informed consent protocol for surveys

El propósito de este protocolo es informarle sobre el proyecto de investigación y solicitarle su consentimiento.
The purpose of this protocol is to inform you about the research project and request your consent.

La presente investigación se titula **"Conocimiento y actitud de los padres sobre la salud bucal en la etapa de dentición primaria en Chachapoyas"** y es elaborada por el proyecto Estomatología de la UNTRM. Este proyecto es dirigido por Nilton B. Rojas Briceño, investigador de la Facultad de Ciencias de la Salud de la UNTRM.
*This research is titled **"Knowledge and attitudes of parents about oral health in the primary dentition stage in Chachapoyas"** and is prepared by the UNTRM Stomatology project. This project is directed by Nilton B. Rojas Briceño, researcher at the UNTRM Faculty of Health Sciences.*

Para ello, se le solicita participar en una encuesta que le tomará **20-30 minutos** de su tiempo. Su participación en la investigación es completamente voluntaria y usted puede decidir interrumpirla en cualquier momento, sin que ello le genere ningún perjuicio. Asimismo, se considera que su participación en este estudio no supone ningún riesgo para usted. Si tuviera alguna consulta sobre la investigación, puede formularla cuando lo estime conveniente durante la encuesta.
*To do this, you are asked to participate in a survey that will take **20-30 minutes** of your time. Your participation in the research is completely voluntary and you can decide to stop at any time, without causing you any harm. Likewise, your participation in this study is considered to pose no risk to you. If you have any questions about the research, you can ask them whenever you consider appropriate during the survey.*

Su identidad será tratada de manera anónima. Asimismo, su información será analizada de manera conjunta con la respuesta de otros encuestados y servirá para la elaboración de artículos y presentaciones académicas. Además, esta será conservada por cinco años, contados desde la publicación de los resultados, en la computadora personal del investigador responsable protegida con contraseña.
Your identity will be treated anonymously. Likewise, your information will be analyzed together with the response of other respondents and will be used to prepare articles and academic presentations. In addition, it will be kept for five years, counted from the publication of the results, on the personal computer of the responsible researcher protected with a password.

Al concluir la investigación, en el correo electrónico que le solicitaremos (no es obligatorio), le enviaremos los resultados del estudio realizado. Asimismo, para consultas sobre aspectos de ética de la investigación, puede comunicarse con la Junta de Revisión Institucional de la Facultad de Ciencias de la Salud de la universidad, al correo facisa@untrm.edu.pe.
At the end of the investigation, in the email that we will request (it is not mandatory), we will send you the results of the study carried out. Likewise, for questions about aspects of research ethics, you can contact the Institutional Review Board of the Faculty of Health Sciences of the university, at facisa@untrm.edu.pe.

Si está de acuerdo con los puntos anteriores, complete los datos y la encuesta a continuación:
If you agree with the above points, please complete the data and survey below:

Fecha/*Date*: ____

Correo electrónico/*Email*: ____

Firma/*Signature*:

SURVEY ON KNOWLEDGE AND ATTITUDE OF PARENTS ABOUT ORAL HEALTH IN THE PRIMARY DENTITION STAGE IN CHACHAPOYAS CITY (PERU)

Instructions

The survey must be completed in its entirety by the student's parent or guardian. Mark with an X inside the brackets you consider and complete with text when you find a line.

PART I: GENERAL INFORMATION

About the parent(s) or guardian(s)

2. The person who completes the survey corresponds to the:

( ) Father ( ) Mother ( ) Male Guardian ( ) Female guardian

3. Age: _________ years

4. Health insurance: ( ) EsSalud ( ) SIS ( ) None

5. Educational level:

( ) Incomplete primary school
( ) Completed primary school
( ) Incomplete secondary school
( ) Completed secondary school
( ) University-level education ⇒ Related to health sciences?: ( )Si ( ) No
( ) Technical-level education ⇒ Related to health sciences?: ( )Si ( ) No
( ) None

About the student

6. Age: _________ years

7. Sex: ( ) Male ( ) Female

8. Health insurance: ( ) EsSalud ( ) SIS ( ) None

9. Grade (in elementary school) or year (in kindergarten) of the student: ____________

10. What is the average monthly income (in soles S/) of your household?

( ) Less than 1000 ( ) 1000–2000 ( ) 2000–3000 ( ) 3000–5000 ( ) More than 5000

PART II: ABOUT STUDENT ORAL HYGIENE

Primary dentition: period of eruption of primary teeth during infancy. **Primary tooth:** primary or childhood tooth that eventually falls out and is replaced by a permanent tooth. **Permanent tooth:** secondary or adult tooth that erupts after a baby tooth falls out and remains in the mouth for the rest of life.

Parent/guardian considerations regarding the student's oral hygiene.

11. Do you consider it important to maintain your child's oral hygiene? ( ) Yes ( ) No
Why? (optional): ____

12. Do you consider it important to treat your child's primary teeth? ( ) Yes ( ) No
Why? (optional): ____

13. Do you consider it important to take your child to the dentist during the primary teething stage?
( ) Yes ( ) No
Why? (optional): ____

14. Do you think that the hygiene of your child's baby teeth affects their permanent teeth? ( ) Yes( ) No
Why? (optional): ____

15. Do you consider it necessary or important to replace the space left by your child's primary tooth?
( ) Yes ( ) No
Why? (optional): ______________________________

16. Do you know devices to maintain the space left by your child's primary tooth? ( ) No ( ) Yes
Indicate which ones (optional): ______________________________

17. Do you know techniques to replace your child's missing primary teeth? ( ) No ( ) Yes
Indicate which ones (optional): ______________________________

Parent's or guardian's knowledge about the student's oral hygiene

18. Which of the following habits of children do you consider to affect their permanent teeth? Select all that apply
( ) Excessive sugar consumption
( ) Nail biting
( ) Teeth grinding or clenching
( ) Thumb sucking
( ) Excessive consumption of acidic foods
( ) Stick out your tongue
( ) Lip biting
( ) Mouth breathing
( ) Cheek biting

19. Which of the following do you recognize as a detrimental effect of previous habits? Select all that apply
( ) Dental caries (progressive destruction of dental tissues due to the action of bacteria and acids)
( ) Dental wear (gradual loss of hard tissue of the teeth)
( ) Temporomandibular joint problems (including jaw pain, difficulty opening or closing the mouth, clicking or popping in the joint, headaches, others)
( ) Pressure on the upper jaws, causing alterations in their growth and position.
( ) Protrusion of upper incisor teeth(upper front teeth are positioned outward in relation to the rest of the teeth and the facial structure)
( ) Palatal arch narrowing (the upper palate, the part of the roof of the mouth, is narrower than normal)

20. Which of the following reasons do you consider for replacing your child's missing baby teeth?
( ) To prevent the movement of other teeth into the empty space left by the baby tooth.
( ) To avoid the mobility of other teeth, not necessarily to the empty space left by the baby tooth.
( ) To provide support for the teeth adjacent to the empty space left by the deciduous tooth.
( ) To help the child to eat correctly

21. Which of the following reasons do you think your child's baby teeth should be treated?
( ) For aesthetics
( ) To eat better
( ) To reinforce the child's confidence
( ) To prevent new caries

22. Does your child currently have a dental problem? ( ) No, turn to 24. ( ) Yes, select all that apply:
( ) Dental caries
( ) Tooth pain and/or sensitivity
( ) Misaligned teeth
( ) Shaking (mobile) teeth
( ) Bleeding gums
( ) Ulcers
( ) Other: ______________________________

23. From the previous question, what is the duration of the oldest dental problem?
( ) One week
( ) Less than 1 month

( ) Less than 6 months
( ) Less than 1 year
( ) More than 1 year

Seeking medical attention from parents or guardians regarding the student's oral hygiene
24. When do you take your child to the dentist?
( ) After the eruption of all permanent teeth
( ) When the child has tooth pain
( ) Without the need for oral treatment
( ) Never

25. How often do you take your child to the dentist?
( ) Once a year ( ) Twice a year ( ) Three times a year ( ) > three times a year ( ) Never

26. What is the most frequent health center for your child's dental care? Select all that apply
( ) Clinic and/or dental office
( ) Public health post
( ) EsSalud
( ) Regional hospital
( ) Lab or dental technician
( ) Pharmacy

27. Do you provide primary care at home for your child's dental discomfort? ( ) No
( ) Yes, What are the attentions?: ______________________________

28. Do you examine your child's teeth from the first year of life?
( ) Every day ( ) Almost every day ( ) Occasionally ( ) Almost never ( ) Never

Student's oral hygiene habits
29. Regarding the student's oral hygiene:
( ) Performed individually by the student
( ) Performed by parent or guardian
( ) Does not perform oral hygiene, skip the following questions

30. Choose the materials to be used during your child's oral hygiene. Select all that apply
( ) Toothbrush ( ) Toothpaste ( ) Dental floss ( ) Mouthwash
( ) Other: ______________________________

31. What method or methods do you use to brush your teeth? Select all that apply
( ) Horizontal ( ) Vertical ( ) Circular

32. How many times a day does your child brush his/her teeth?
( ) One ( ) Two ( ) Three ( ) More than three

33. At what times of the day does the student brush his teeth? Select all that apply
( ) Upon waking and rising ( ) After each meal ( ) Before sleeping ( ) Other:

34. How often does the student change toothbrushes?
( ) Each month ( ) 2 to 3 months ( ) 4 to 6 months ( ) More than 6 months
